# Anthropogenic Pressures, Rather Than Plant Vigour, Promote Insect Herbivory Rates on *Securidaca longepedunculata* Along Elevation in a South African Woodland

**DOI:** 10.1002/ece3.73363

**Published:** 2026-04-08

**Authors:** Mashudu Patience Mamathaba, Bopaki Phogole, Orou G. Gaoue, Kowiyou Yessoufou

**Affiliations:** ^1^ Department of Geography Environmental Management and Energy Studies, University of Johannesburg, Auckland Park Kingsway Campus South Africa; ^2^ African Centre for DNA Barcoding, Department of Botany and Plant Biotechnology University of Johannesburg South Africa; ^3^ Department of Ecology and Evolutionary Biology University of Tennessee Knoxville Tennessee USA; ^4^ Faculty of Agronomy, University of Parakou Parakou Benin

**Keywords:** anthropogenic disturbances, environmental disturbances, herbivory, medicinal plants, plant vigour hypothesis, proximity to human settlement, root harvesting

## Abstract

Understanding why some plants experience greater herbivory than others is central to predicting population dynamics and ecosystem resilience. We tested the plant vigour hypothesis, the resource concentration hypothesis and the role of anthropogenic disturbance in shaping herbivory of a heavily harvested medicinal plant in South Africa. Plant height and canopy size were positively associated, indicating coordinated growth, yet these traits responded differently to environmental gradients: Larger canopies were more common at higher elevations and closer to human settlements, whereas taller plants occurred at lower elevations and farther from settlements. Root harvesting was negatively size‐dependent, with taller individuals harvested less intensively; however, harvesting pressure increased significantly at lower elevations and near settlements, reflecting strong disturbance effects. Insect herbivory increased with harvesting intensity but was unrelated to plant vigour, providing little support for the plant vigour hypothesis in this system. We suggest that anthropogenic pressures may increase herbivory through multiple pathways, including increased plant exposure following disturbance, stress‐induced reductions in plant defence and potential disruption of plant–insect dynamics. Cumulatively, our findings show that herbivory risk is structured more strongly by anthropogenic disturbance and spatial environmental gradients than by intrinsic plant vigour, highlighting the interactive roles of ecological and human drivers in shaping plant–insect dynamics.

## Graphical abstract

Understanding how chronic anthropogenic disturbances shape species interactions can enhance our ability to predict biodiversity resilience and guide conservation actions. Our study reveals that human disturbance shapes herbivory.

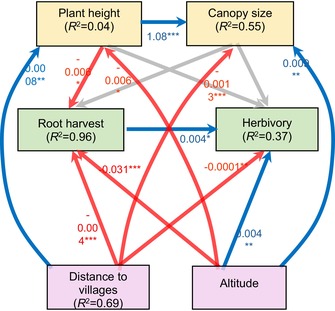

## 1 Introduction

South Africa is home for ~24,000 plant species (Mamathaba et al. 2022), several of which are medicinally valued plants across Africa, for example, *Securidaca longepedunculata Frese* (Tshisikhawe 2002). *S. longepedunculata* (Polygalaceae) bears leaves of various sizes with an above‐ground stem of small to medium‐size (Van Wyk et al. 2009; Mustapha 2013). It is found in subtropical hot and humid regions in semi‐arid scrub to deep forest and woodland habitats in Africa (Moeng 2010; Tabuti et al. 2012).

Specifically in South Africa, *S. longependuculata* is distributed in the Northwest and Limpopo provinces (Tshisikhawe 2002). Unfortunately, the species faces significant threats mainly due to an unsustainable harvesting of its stem/bark and mostly roots for medicinal purposes, which hinders its survival (Mukhithi 2023), causing a noticeable decline of its already limited populations and forcing decision‐makers to categorise it as a threatened and protected species (Mukhithi 2023). It is well established that harvesting not only affects reproduction but also alters plant growth, thus compromising the species' long‐term viability (Ticktin 2004; Gaoue and Ticktin 2010; Oni et al. 2014). Despite its protected status, its stem, bark and roots remain heavily traded in South Africa (Mukhithi 2023) mainly for its aphrodisiac property (Tshisikhawe 2002; Moeng 2010) and this is also reported in other African countries (Mustapha 2013; Tabuti et al. 2012). This anthropogenic pressure, that is, extirpation of the plant from ecosystem, has consequences on biotic interactions, for example, reduction of resource availability for herbivorous insects.

Herbivores, indeed, can shape ecosystem processes and functions. Plant species targeted by insect herbivores are generally rich in nutrients (e.g., nitrogen and phosphorus), which are important elements for insect physiology (Gu et al. 2022; Rode et al. 2017). In the meantime, other plant species are avoided by herbivores because they are well‐defended and rich in secondary compounds and leaf dry matter content, which make digestion complex (Singh et al. 2022; Roeder et al. 2019).

Due to their feeding behaviour, insect herbivores are actively involved in the transfer of energy across terrestrial ecosystems, thus influencing not only plant growth and fitness but also shaping ecosystem dynamics (Ancheta and Heard 2011; Belovsky and Slade 2000; Kristensen et al. 2020; Agrawal and Maron 2022). For example, insect herbivores consume on average 15%–30% of plant biomass (Cyr and Pace 1993; Coupe and Cahill Jr 2003; Hambäck and Beckerman 2003; Hartley and Gange 2009), although the intensity of this consumption is biome‐dependent, varying from 7.1% in temperate forests to ~10% in tropical forest (Coley and Barone 1996) and 50% in Neotropical savannas (Lopes and Vasconcelos 2011).

However, despite the ecological importance of insect herbivory, it receives less attention in several regions and ecosystems (Liu et al. 2024). Interestingly, large variations in the intensity of leaf herbivory were reported among and within species (Zhang et al. 2016; The Herbivory Variability Network 2023), raising questions about the drivers of these large variations. The intensity of insect herbivory is influenced by environmental (e.g., altitude), biological, for example, plant functional traits (height, canopy size) and anthropogenic activities (plant harvest and proximity to human settlement). For example, herbivory rates are typically low at high altitudes but significantly higher at low altitudes (Sam et al. 2015), and this is largely habitat‐specific (Dostálek et al. 2018). Firstly, herbivore damage tends to be greater in open habitats than in forests (Dostálek et al. 2018). Secondly, the extent of biomass removal by herbivory can change with environmental conditions. For example, previous studies reported that herbivory rates are greater at low altitudes than at high altitudes in forest habitats, whereas no differences in herbivory rates were found between populations at different altitudes in open habitats (Dostálek et al. 2018). The significant difference in herbivory rates at different altitudes and habitats is related to differential investment in plant defence across altitudes. Plants at high altitudes in forests are less susceptible to their attacks because these plants are usually less palatable and are generally small (Cui et al. 2021; Dostálek et al. 2018; Moreira et al. 2018).

Furthermore, the plant vigour hypothesis (Price 1991) predicts that insect herbivores would predominantly select bigger and more vigorously growing plants (Cornelissen et al. 2008), resulting in higher herbivory rates in larger plants (Schlinkert et al. 2015; but see The Herbivory Variability Network 2023). This hypothesis is grounded on the assumption that larger plants offer increased quantity and range of resources for herbivores. In this hypothesis, vigour referred to any plant growing faster and thus exhibiting larger size (Gonçalves‐Alvim et al. 1999). Plant height and canopy size are often used as a proxy for plant vigour (Zhang et al. 2023). Thus, we expect taller plants and plants with larger canopy to suffer higher insect herbivory (see also Yuan and Wang 2025).

Finally, anthropogenic disturbance can also influence insect herbivory (Pablo‐Rodríguez et al. 2023). However, the intensity of insect herbivory depends on the insect's feeding guild. For example, while generalist insect herbivores maintain a high level of herbivory irrespective of disturbance level (Tscharntke et al. 2002; Tscharntke and Brandl 2004), herbivory by specialists declines substantially when their host plants are, for example, lethally harvested by human (Novotny et al. 2010). Several hypotheses predict that herbivory is contingent on the population density of host plants. For example, the resource concentration hypothesis predicts that specialist insect herbivory is expected to increase with increasing plant population density because high‐density populations have a higher availability of resources (Root 1973; Sholes 2008; but see Sadiki et al. 2024). Under the resource availability hypothesis, we expect herbivory to be low in heavily root‐harvested populations. *S. longepedunculata* plants experience several stresses due to environmental (altitude), and anthropogenic (lethal harvest by humans) factors in addition to insect herbivory, especially during the flowering season (Tshisikhawe et al. 2012). As such, any agent of changes in plant density would influence insect herbivory. Such agents may be humans, especially when they lethally harvest plants, for example, root harvest, for their use.

In the present study, we investigate how multiple stressors (environmental and anthropogenic) shape insect herbivory in S. *longepedunculata*. We hypothesised herbivory rates will be lower in heavily root‐harvested populations due to lower resource availability and subsequent vigorous chemical and physical antiherbivory defence consistent with the resource availability hypothesis. We also hypothesised that taller plants and plants with larger canopy will suffer lower insect herbivory consistent with the life history theory, which predicts negative size‐dependent herbivory to protect costly biomass produced over a longer time period.

## 2 Methodology

### 2.1 Study System

**FIGURE 1:**
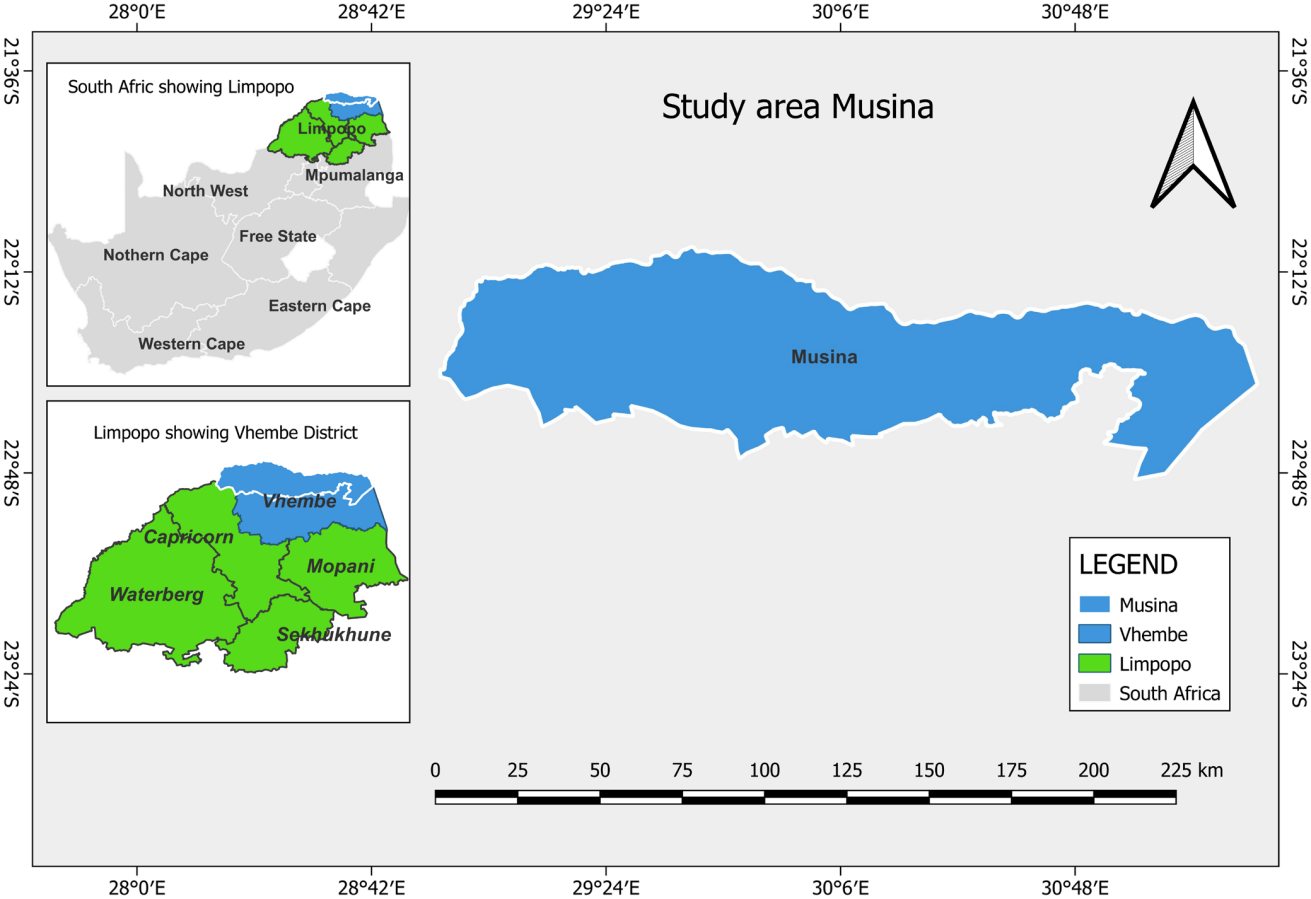
Geographic location of our study area.

This study was carried out in the *Muswodi* region of Limpopo province (23.40.13° S, 29.41.79° E) in the northernmost part of South Africa (Figure 1). The Limpopo province is dominated by the savannah grassland biome, which is known in the region as bushveld (Pieterse et al. 2020; Smit 2004). Limpopo experiences almost no rain and low humidity during the winter season, while summers are extremely hot and humid, usually with afternoon thunderstorms (Rapolaki 2020; Sikhwari 2019). The basin's average precipitation ranges from 200 mm per year in hot and dry areas to 1500 mm per year in higher rainfall areas (Rapolaki et al. 2020; Zhu and Ringler 2010). Peak temperatures regularly exceed 40°C despite average daytime temperatures of around 33°C (Rapolaki et al. 2020; Zhu and Ringler 2010).

*Securidaca longepedunculata*, known as the violet tree, or commonly known as *Mpesu* by the *Vhavenda* people in South Africa, is a medicinal plant species (Oni et al. 2014), widespread in Africa and found in South Africa's North‐West and Limpopo provinces (Tshisikhawe et al. 2012). It grows from 550 m to 1800 m above sea level in woodland and arid savanna soils, and it can be found on either sandy, acidic, or rocky soils. Its habitat varies with climate, from hot, arid to humid climates, and in broad vegetation ranges, from semi‐arid to dense forest (Tshisikhawe et al. 2012). The violet tree is a small to medium‐sized tree and can reach a height of 6 m (Mustapha 2013; Van Wyk et al. 2009) with a distinctive pale grey bark.

### 2.2 Data Collection

In the Limpopo province, South Africa, we identified three populations of *S. longepedunculata* occurring in woodland forests in three villages, namely *Mutale*, *Tshirunzhini* and *Gundani*. In each population, we set up 15 m × 15 m plots along a north–south transect such that two consecutive plots are separated by 100 m. In each plot, all individuals of *S. longepedunculata* were tagged with numbered aluminium tags and mapped in local *x‐y* coordinates.

Data were collected in all populations as detailed below. The complete dataset is presented in Table S1 and available publicly at Figshare (Mamathaba et al. 2025).

#### 2.2.1 Environmental Variable

We determined the altitude corresponding to the location of each individual of *S. longepedunculata* using MyALTITUDE V 2.8.0 (Dayana Networks Ltd., Vancouer, Albeta, Canada) digital application.

#### 2.2.2 Functional Traits

We used a tape meter to measure the height of each individual *S. longepedunculata* within a plot, and a clinometer was utilised when an individual was taller than 1 m. Furthermore, we measured the canopy size of each plant in the plot. This was done by taking two diameter measurements of the canopy, the first following a north–south direction and the second following an east–west direction. Then, the average of these two diameters was used to estimate the canopy size as the area of a circle.

#### 2.2.3 Anthropogenic Disturbance

The anthropogenic disturbance was measured in two ways: firstly, as root harvest intensity and secondly, as the distance of a plot to the three human settlements in the study area. Distance to settlement was calculated from the centroid of each vegetation plot to the centroid of the nearest settlement. Root harvest intensity was determined by simply counting the number of holes found in a plot and attested by a local traditional healer as corresponding to a place where an individual of *S. longepedunculata* has been physically removed. The field data collection was done entirely with the traditional healer. A hole was validated as corresponding to a harvested individual of *S. longepedunculata* based on two criteria defined by the traditional healer: (i) his years of experience with root harvesting of *S. longepedunculata*, and (ii) the presence in the hole of the remainder of the harvested root or an observed new shoot of *S. longepedunculata* springing from the hole. The distance of the plot to the human settlement (Mutale, Tshirunzhini, Gundani) was determined using Google Earth.

#### 2.2.4 Herbivory

In a plot, we randomly collected 10 leaves from each individual of *S. longepedunculata*. We used *LeafByte* to estimate the proportion of each leaf eaten by insects (Getman‐Pickering and Campbell 2019). An average estimate of herbivory per individual was calculated.

### 2.3 Data Analysis

Prior to modelling, model assumptions were verified using residual plots and normality diagnostics, and where assumptions were violated, appropriate transformations were applied. All models were fitted as mixed‐effects models, with site (Mutale, Tshirunzhini, and Gundani) included as a random effect to account for spatial clustering. Because herbivory was measured as proportional data, it was arcsine square‐root transformed prior to analysis and fitted using a linear mixed‐effects model (function lmer). Root harvest intensity, measured as count data, was analysed using a negative binomial generalised linear mixed‐effects model (glmer.nb) to account for overdispersion. Canopy size and plant height were log‐transformed to improve normality and fitted using linear mixed‐effects models (lmer).

Our primary response variable was herbivory, quantified as the proportion of leaf area damaged per individual plant. Herbivory was modelled as a function of altitude (Sam et al. 2015; Sadiki et al. 2024), plant height and canopy size (reflecting the plant vigour hypothesis; Price 1991), and anthropogenic disturbance (Barreto et al. 2021; Pablo‐Rodríguez et al. 2023). Anthropogenic disturbance was operationalised as (i) root harvest intensity and (ii) distance from the plant population to the nearest human settlement.

We then modelled root harvest intensity as a function of plant height, canopy size, altitude, and distance to the nearest settlement. Taller plants and individuals with larger canopies were expected to be more detectable to harvesters. We further predicted higher harvesting intensity at lower altitudes, where terrain is more accessible, and in closer proximity to settlements, where harvesting pressure is likely greater.

Next, canopy size was modelled as a function of plant height, altitude and distance to settlements. Because plant height and canopy size are structurally related (Sadiki et al. 2024), a positive association was expected. We also tested whether canopy size varied along elevational gradients and whether proximity to settlements—used as a proxy for disturbance—was associated with reduced canopy development due to stress or biomass removal.

Finally, plant height was modelled as a function of altitude (Mao et al. 2018) and distance to settlements, given that repeated harvesting and disturbance may limit plant growth (Rolff and Ågren 1999).

To evaluate direct and indirect pathways simultaneously, all component models were integrated into a piecewise structural equation model (SEM) and analysed using the psem function in the R package piecewiseSEM (Lefcheck 2016).

All analyses were conducted in R version 4.1.2 (R Core Team 2015). The full R scripts are provided in Supporting Information (SI2) and archived on Figshare (Mamathaba et al. 2025).

## 3 Results

**FIGURE 2:**
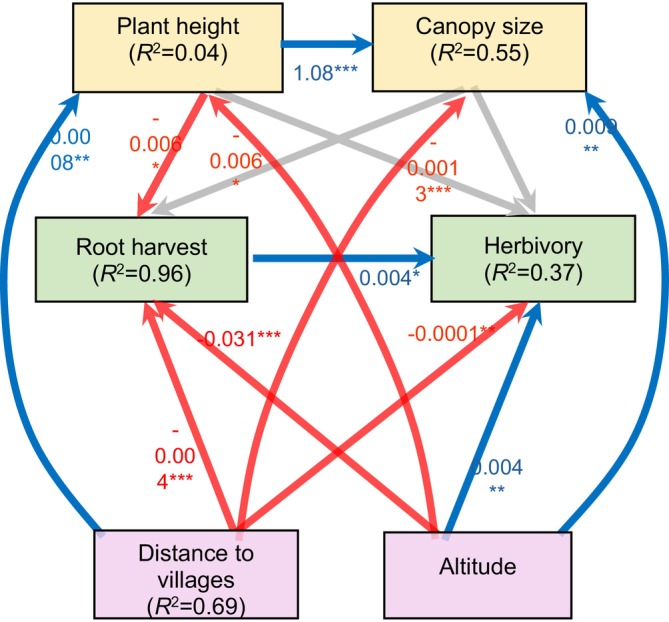
Structural equation model illustrating the direct and indirect relationships between functional traits (height, canopy size), environmental factors (altitude), anthropogenic pressures (root harvest, distance to villages) and insect herbivory in *Securidaca longepedunculata* in South Africa. While plant height and canopy size were positively associated, they responded differently to elevation and distance to villages—taller plants were found at lower altitudes and farther from human settlements, whereas large‐canopy plants occurred at higher altitudes and closer to human settlements. Grey arrows indicate non‐significant paths. Conditional *R* ^2^ (reporting the variance explained by both fixed and random factors) is reported on each variable. Asterisks on path coefficients indicate significance level: *p < 0.05, **p < 0.001 and ***p < 0.0001.

**FIGURE 3:**
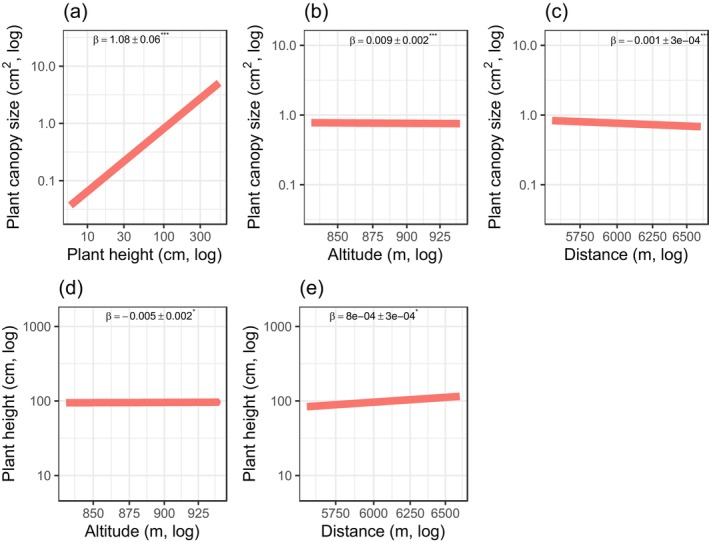
Bivariate relationships between plant height, canopy size, altitude and proximity to human settlements in *Securidaca longepedunculata*. Taller plants were associated with larger canopy sizes (a). Large‐canopy plants were more common at higher altitudes (b) and closer to villages (c). However, taller plants were found at lower altitudes (d) and tended to be located farther from villages (e). Asterisks on path coefficients indicate significance level: *p < 0.05, **p < 0.001 and ***p < 0.0001.

Both significant and nonsignificant paths included *R* ^2^ values are summarised in Figure 2. First, our results linked the height of *S. longepedunculata* to canopy, altitude, and distance to human settlement (Figure 2). Plant height and canopy size were positively associated but responded differently to elevational gradient and proximity to human settlements (Figure 2). Specifically, we found that taller *S. longepedunculata* plants tended to have larger canopy size (*β* = 1.08 ± 0.06, *p* < 0.001, Figure 3a), and large canopy plants were mostly found at higher altitudes (*β* = 0.009 ± 0.002, *p* = 0.001, Figure 3b) and closer to human settlements (*β* = −0.001 ± 0.0003, *p* < 0.001, Figure 3c). In contrast, taller *S. longepedunculata* plants were found at lower altitudes (*β* = −0.005 ± 0.002, *p* = 0.045, Figure 3d) and located mostly away from human settlement (*β* = −0.00000008 ± 0.0003, *p* = 0.0228, Figure 3e).

**FIGURE 4:**
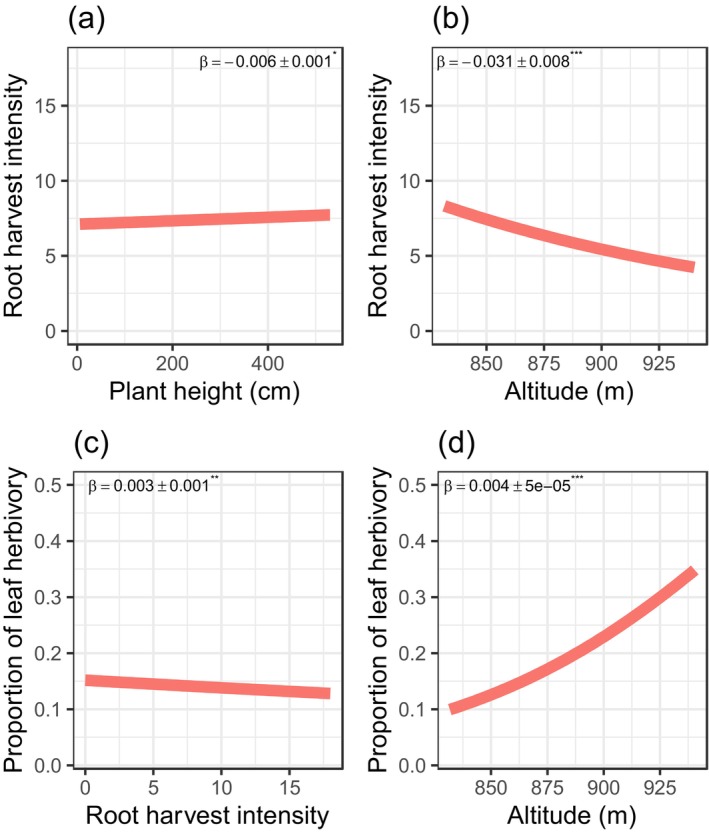
Bivariate relationships between root harvesting, plant height, altitude, proximity to human settlements, and insect herbivory in *Securidaca longepedunculata*. Negative size‐dependent root harvesting, with taller plants experiencing lower harvest intensity (a). However, root harvest was more intense at lower altitudes (b). Insect herbivory increased with root harvest intensity (c) and at higher altitudes (d). Asterisks on path coefficients indicate significance level: *p < 0.05, **p < 0.001 and ***p < 0.0001.

Second, we found negative size‐dependent root harvesting with taller *S. longepedunculata* harvested at a lower intensity (*β* = −0.006 ± 0.001 *p* = 0.001, Figure 4a), but root harvest was intense at lower altitudes (*β* = −0.031 ± 0.008, *p* = 0.0003, Figure 4b) and closer to human settlements (*β* = −0.004 ± 0.001, *p* = 0.018, Figure 3).

Finally, insect herbivory increased with root harvest intensities (*β* = 0.003 ± 0.001, *p* = 0.01, Figure 4c), but also with proximity to human settlement (*β* = −0.0001 ± 0.0001, *p* = 0.004, Figure 3) and altitude (*β* = 0.004 ± 0.00005, *p* < 0.001, Figure 4d).

## 4 Discussion

### 4.1 Plant Vigour Hypothesis Does Not Explain Leaf Herbivory

Plant vigour hypothesis predicts that insect herbivores predominantly select bigger and more vigorously growing plants as their primary resources (Price 1991). Evidence for this was reported in various studies. For example, Schlinkert et al. (2015) observed higher herbivory rates on larger plants. However, contrasting findings are also reported showing negative relationships between plant size (height or canopy size) and herbivory (Nakamura et al. 2017), perhaps because larger trees invest heavily in chemical defences, limiting herbivore–plant interactions (Whitman 2017; Karban 2020). In the present study, we found no support for plant vigour hypothesis as plant vigour, approximated by plant height and canopy size, did not correlate with herbivory intensity. We propose that the link between plant vigour and herbivory may be disrupted by various anthropogenic pressures that individuals of *S. longepedunculata* experience in the study area, including lethal root harvesting. Such disturbance may independently increase plant investment in chemical defence, which can limit herbivory. This warrants further empirical testing. However, recent studies show that damage to roots can induce vigorous defence mechanisms, particularly for long‐lived species that are expected to invest in root biomass and defence, consistent with the optimal defence theory (Rasmann and Agrawal 2008; Xiao et al. 2019).

### 4.2 Anthropogenic Pressures Explain Insect Leaf Herbivory

Anthropogenic pressures were approximated by integrating site proximity to human settlements and root harvesting, which can directly kill *S. longepedunculata* plants. Both proxies show opposite effects on herbivory. First, we found higher leaf herbivory rates closer to human settlements. This may imply that *S. longepedunculata* populations that are closer to human settlements are easily accessible, making anthropogenic pressures (e.g., root harvesting) occur more frequently. This also suggests that *S. longepedunculata* populations under anthropogenic pressures are more vulnerable to herbivory. If that is the case, we would expect a positive relationship between root harvesting and herbivory. Our finding supported this relationship such that populations of *S. longepedunculata* that suffer heavy root harvesting suffer higher herbivory. Several scenarios are plausible to explain our findings.

First, as reported in early studies, insect herbivores in the tropics are mostly generalist (see Novotny et al. 2010) which tend to maintain consistently high levels of herbivory across disturbed landscapes (Tscharntke et al. 2002; Tscharntke and Brandl 2004). Second, root harvest‐related increased mortality of *S. longepedunculata* plants may lead herbivores to rely more heavily on the remaining unharvested plants. Third, our findings challenge conventional expectations, as lethal root harvesting is expected to reduce resource availability (see Novotny et al. 2010) and thereby lower herbivory as predicted by the resource concentration hypothesis (Root 1973; Kéry et al. 2001; Ostergard and Ehrlén 2005; Sholes 2008). The resource concentration hypothesis posits that herbivory is amplified in areas of high host plant density, which is expected to offer abundant resources (e.g., leaves, seeds, etc.) (Root 1973; Sholes 2008; but see Grez and Gonzalez 1995 and Sadiki et al. 2024). Humans, through lethal root harvesting, reduce the density of *S. longepedunculata* and as such likely trigger compensatory re‐growth of *S. longepedunculata*, which offset the loss of individuals caused by lethal harvest. Indeed, we observed, during field data collection, instances of new shoots emerging from stem fragments left by harvesters, suggesting potential resilience mechanisms that warrant further study. Our findings, therefore, suggest that lethal root harvest, rather than simply suppress insect–plant interactions, may drive complex feedback mechanisms that maintain high activities of insect herbivores. Finally, alteration of predator–prey dynamics due to anthropogenic disturbance may deregulate the top‐down control of natural enemies on herbivores, allowing herbivore populations to thrive, leading to higher levels of herbivory (Santamaría et al. 2022; Sentis et al. 2022; Le Roux et al. 2019; Jia et al. 2018).

Overall, stressors are known to drive significant declines in plant performance (Villani and Wright 1990; Blossey and Hunt‐Joshi 2003; Blackshaw and Kerry 2008; Zvereva and Kozlov 2012). Our findings indicate that anthropogenic pressures may increase herbivory, which cumulatively could negatively affect plant performance and population dynamics. We therefore call for further research on how root harvesting influences plant population dynamics, and how that shapes insect herbivores' behaviour, and broader ecological processes across diverse ecosystems. Understanding these relationships is critical for mitigating the unintended consequences of lethal root harvesting on biodiversity and ecosystem function.

### 4.3 Herbivory Is High at High Elevation

Our findings further indicate intense activities of insect herbivores at high elevations. This could be due to processes consistent with the resource concentration hypothesis and also weak chemical defence at high elevations. If resources, for example, leaves, are concentrated on plants at high elevation, we would expect a high density of insect herbivores and, therefore, high herbivory intensity at high elevation (resource concentration hypothesis; Kéry et al. 2001; Ostergard and Ehrlén 2005; Root 1973; Sholes 2008). Although we did not specifically investigate the number of leaves along elevation, we did find that individuals of *S. longepedunculata* tend to be shorter but develop larger canopies at high elevations. Such plant architecture at high elevation implies richness in leaves (e.g., Sadiki et al. 2024), which, therefore, may potentially drive a high density of herbivores, thus high herbivory as predicted in the resource concentration hypothesis. It is important to note that contradicting evidence for the hypothesis is also reported (e.g., Grez and Gonzalez 1995; Sadiki et al. 2024), implying the need for further tests for *S. longepedunculata* for clarification.

Furthermore, it is equally important to note that elevation is an environmental stressor to plants, and as such, plants at high elevation may preferentially invest in overcoming stresses imposed by elevation at the expense of investing in chemical defences against herbivores. Consequently, plants at high elevations may be more vulnerable to intense activities of herbivorous insects as predicted in the elevational herbivory defence hypothesis (Pellissier et al. 2014; Rasmann et al. 2014), resulting in the high herbivory we found at high elevation. There are several reports on plants relaxing their defences against herbivores at high elevations (e.g., Pellissier et al. 2014; Callis‐Duehl et al. 2017). Callis‐Duehl et al. (2017) reported a decline in plant physiochemical defence at high elevations, including low specific leaf area, which is indicative of slow turnover in leaf production at high elevations. Such low turnover may predispose the few existing leaves to high herbivory, given that high elevation plants are less resistant to herbivory (Pellissier et al. 2014; but see Rasmann et al. 2014 and Wu et al. 2021).

It is important to highlight some plant‐environmental interactions in our study that are counter‐intuitive. For example, plant height correlates positively with canopy size but canopy size is positively associated with altitude whereas plant height is negatively associated with altitude. This apparent inconsistency arises from the fact that these relationships are conditional relationships (i.e., estimated while holding other variables constant) estimated within a multivariate framework (Structural Equation Model), rather than simple bivariate correlations. First, while plant height and canopy size are positively associated overall (i.e., taller plants tend to have larger canopies), altitude affects each trait differently after accounting for their covariance. Specifically, (i) altitude is negatively associated with plant height (plants tend to be shorter at higher elevations), but (ii) altitude is positively associated with canopy size *after controlling for plant height*. This means that, although taller plants generally have larger canopies, high‐elevation plants are structurally shorter but tend to allocate proportionally more growth to lateral expansion (canopy development) relative to vertical growth. In other words, altitude influences plant architecture, not just overall size. Our structural equation model disentangles these direct effects, which may differ in direction despite the positive correlation between height and canopy. A similar rationale applies to distance from settlements. Taller plants were more common further from settlements, suggesting reduced disturbance effects on vertical growth. However, canopy size showed a different conditional response once plant height was accounted for. These patterns likely reflect disturbance‐mediated shifts in growth form rather than simple size gradients. Critically, these results are not contradictory statistically. In multivariate systems, a predictor can have (i) a positive effect on one trait, but negative effect on another correlated trait, while those traits themselves remain positively correlated. Such outcomes are common when environmental gradients influence resource allocation strategies or plant architecture, rather than overall vigour alone.

## 5 Conclusion

*Securidaca longepedunculata* is a widespread plant species across Africa. It is of high medicinal value, especially its roots, resulting in heavy anthropogenic pressures. Unfortunately, the search for its roots is the most common harvest approach used in South Africa, and this leads to the extirpation of individuals from the ecosystems, thus posing a risk to the future of this species. Our findings indicate that populations of *S. longepedunculata* closer to human settlements are most vulnerable to anthropogenic pressures, and these pressures seem to promote insect herbivory, which further adds to the stress of the populations. Our findings further indicate that herbivory is intense at high elevations. One of the plausible explanations is that perhaps more resources are available for herbivorous insects at high elevations, following the resource concentration hypothesis. Also, traditionally, we expect herbivory pressure to be intense at low elevations, resulting in plants at lower elevations to invest more in their defences (elevational herbivory defence hypothesis; Pellissier et al. 2014; Rasmann et al. 2014). Mixed findings were reported for this hypothesis (see Descombes et al. 2017 for negative, Moreira et al. 2018 for positive and Alonso‐Amelot et al. 2007 for lack of relationship between elevation and defence). We found for *S. longepedunculata* an intense herbivory rather at high elevations. Our finding matches the general trend reported recently, which revealed out of 6262 angiosperm species, the highest proportion at high elevations of plants with glandular trichomes—known for their contributions to chemical and physical defences against herbivorous insects (Wu et al. 2021).

Overall, our study reveals the extent of a panoply of stressors (humans, herbivory, elevation) that constrain the dynamics of the populations of *S. longepedunculata*. Our findings imply that root harvest may have secondary negative effects mediated through herbivory which cumulatively with root harvesting and environmental stressors may be driving the population decline of *S. longepedunculata*. This is key knowledge with which to inform the management of human interactions with this valued medicinal plant. Such management requires population dynamics studies of the species to identify which demographic stages may have negligible impacts on the population dynamics of *S. longepedunculata*. People may be allowed to harvest individuals at such stages for their medicinal uses.

## Author Contributions

**Mashudu Patience Mamathaba:** data curation (lead), formal analysis (lead), methodology (equal), writing – original draft (lead). **Bopaki Phogole:** data curation (equal), formal analysis (supporting), methodology (supporting), project administration (supporting), resources (supporting). **Orou G. Gaoue:** conceptualization (equal), formal analysis (equal), methodology (equal), supervision (equal), validation (equal), visualization (equal), writing – review and editing (equal). **Kowiyou Yessoufou:** conceptualization (lead), formal analysis (equal), funding acquisition (lead), investigation (lead), methodology (lead), project administration (lead), resources (lead), supervision (lead), validation (lead), visualization (lead), writing – review and editing (lead).

## Funding

This work was supported by the Fulbright US Scholar, PS00241633, National Science Foundation, 2107127 and National Research Foundation, SRUG22051210107.

## Conflicts of Interest

The authors declare no conflicts of interest.

## Supporting information


**Data S1:** ece373363‐sup‐0001‐Supinfo1.txt.

**Data S2:** ece373363‐sup‐0002‐Supinfo2.pdf.

**Table S1:** ece373363‐sup‐0003‐TableS1.xls.

## Data Availability

Data and R script used for the statistical analyses are publicly published in an open‐access data repository on FigShare (Mamathaba et al. 2025; https://doi.org/10.6084/m9.figshare.28357310).
